# Predicting the influence of particle size on the glass transition temperature and viscosity of secondary organic material

**DOI:** 10.1038/s41598-020-71490-0

**Published:** 2020-09-16

**Authors:** Markus Petters, Sabin Kasparoglu

**Affiliations:** Department of Marine, Earth, and Atmospheric Sciences, NC State University, Raleigh, 27695-8208 USA

**Keywords:** Atmospheric science, Atmospheric chemistry

## Abstract

Atmospheric aerosols can assume liquid, amorphous semi-solid or glassy, and crystalline phase states. Particle phase state plays a critical role in understanding and predicting aerosol impacts on human health, visibility, cloud formation, and climate. Melting point depression increases with decreasing particle diameter and is predicted by the Gibbs–Thompson relationship. This work reviews existing data on the melting point depression to constrain a simple parameterization of the process. The parameter $\xi$ describes the degree to which particle size lowers the melting point and is found to vary between 300 and 1800 K nm for a wide range of particle compositions. The parameterization is used together with existing frameworks for modeling the temperature and RH dependence of viscosity to predict the influence of particle size on the glass transition temperature and viscosity of secondary organic aerosol formed from the oxidation of $\alpha$-pinene. Literature data are broadly consistent with the predictions. The model predicts a sharp decrease in viscosity for particles less than 100 nm in diameter. It is computationally efficient and suitable for inclusion in models to evaluate the potential influence of the phase change on atmospheric processes. New experimental data of the size-dependence of particle viscosity for atmospheric aerosol mimics are needed to thoroughly validate the predictions.

## Introduction

Atmospheric aerosols play a central role in atmospheric chemistry by providing surface and volume for multiphase reactions to proceed. Crystalline solid or amorphous viscous particles contain little or no water. At moderate relative humidity (RH) some particles form concentrated aqueous solutions, and at high RH ($>\,80\%$) particle-bound water may become the dominant aerosol component^1^. Diffusion of gases through solids and intraparticle mixing times in solids are slow. Thus, chemical reactions within the bulk of a solid particle may be slow (or effectively impossible)^2–5^. Similarly, absorptive gas-particle partitioning is predicated on the existence of a well-mixed liquid organic phase. When the organic phase is viscous, gas-particle equilibration time scales of semi-volatile organic compounds can become long ($>>1\,\hbox {h}$), thus necessitating kinetic instead of thermodynamic frameworks to model secondary organic aerosol formation^6–9^. Measured equilibration time scales for water in viscous and glassy solids are fast at warmer temperatures ($T>- 10\,^{\circ } \hbox {C}$)^10–13^. In turn, viscosity exerts little influence on atmospheric processes involving water uptake. However, at upper-free tropospheric temperatures ($T<-40\,^{\circ } \hbox {C}$), water uptake is slow^14^. This leaves solid surfaces partially or fully intact for considerable duration at humidities exceeding ice saturation. Some of these surfaces may serve as heterogeneous ice nucleation sites^11,15–17^ and thus may influence ice crystal concentration in cirrus clouds. The above mentioned effects imply that particle phase state plays a critical role in understanding and predicting aerosol impacts on human health, visibility, cloud formation, and climate^18^.

Particle viscosity can be used to categorize the amorphous particle phase state into liquid ($10^{-3}\,\hbox {Pa}\,\hbox {s}$, “water” to $10^{4}\,\hbox {Pa}\,\hbox {s}$, “peanut butter”), semi-solid ($10^{4}$–$10^{12}\,\hbox {Pa}\,\hbox {s}$, “glass”), and glassy solid ($>10^{12}\,\hbox {Pa}\,\hbox {s}$) regimes. Particle viscosity sharply increases with decreasing temperature^19,20^. A common convention is to equate the temperature at which the viscosity reaches $10^{12}\,\hbox {Pa}\,\hbox {s}$ with the glass transition temperature that is obtained using differential scanning calorimetery^21^. Zobrist et al.^22^ first suggested that organic particles might form glassy states at low RH and cold temperatures. Soon after, Virtanen et al.^23^ showed that secondary organic aerosol (SOA) generated in plant-chambers and atmospheric organic aerosol (OA) can exist in solid states. This was inferred from “bounce factor” measurements, where the bounce factor refers to the fraction of particles that rebound upon impaction on a surface. Particles that deform upon impaction are more likely to adhere to the surface than solid particles; larger bounce factors thus indicate a more solid phase state.

The initial discovery that ambient OA particles bounce implies that they are semi-solid at room temperature. A large number of follow-up studies investigated viscosity of ambient organic aerosol and environmental chamber aerosol. These studies were recently reviewed by Reid et al.^18^. The main thrusts described therein are briefly summarized here. A number of new techniques were developed to better quantify aerosol phase state^24–30^. Factors that increase OA viscosity have been identified to be increasing compound molecular weight^31^, O:C ratio^32^, and functional group composition^19,33^. Viscosity and volatility are inversely correlated^19,34,35^. Under dry conditions and at room temperature many secondary organic aerosols are semi-solid. The extent of the reduction in viscosity with RH depends on the hygroscopicity of the aerosol^29,36–38^. Monoterpenes and aromatic precursors produce more viscous particles than isoprene SOA^27,30,37,39,40^. Ambient SOA phase state follows a diurnal cycle, with higher viscosity observed during nightime^41^. Reactive uptake has been shown to create viscous shells or cores^42,43^. Under humid conditions water uptake rapidly reduces viscosity. Measurements and models quantifying the amorphous phase state diagram over a wide range of temperature and humidity have been developed^16,22,31,40,44–46^. The relationships between viscosity and diffusion-coefficients, to first order predicted by the Stokes–Einstein equation, have been clarified. The Stokes–Einstein equation holds for predicting the diffusion of larger organic molecules, but errs for small molecules such as water^2,9–11,13,47,48^. First estimates of the prevalence of gassy organic states in the atmosphere have been made^19,32,49^. The potential dependence of viscosity on particle size has received less attention.

As particle size decreases, the surface to volume ratio increases and a number of aerosol physicochemical properties are affected. Due to surface tension, the vapor pressure over a curved particle increases (Kelvin effect) and melting point decreases (Gibbs–Thompson effect). Surface tension itself increases with decreasing particle diameter below the Tolman length^50,51^. Furthermore with decreasing particle diameter, the deliquescence relative humidity and efflorescence relative humidity increases^52–54^, the equilibrium hygroscopic growth factor decreases^55^, the condensation coefficient of the gas molecule to the particle surface decreases^56^, and liquid–liquid phase separation is suppressed^57–60^. Virtanen et al.^23,61^ showed that the bounce factor of organic particles produced by OH dominated oxidation of pine emitted volatile organic compounds sharply decreases for particles with $D<30\,\hbox {nm}$, but not for solid ammonium sulfate particles. This implies either that composition for $D<30\,\hbox {nm}$ SOA differs from those of the larger particles, or that size influences particle viscosity. Cheng et al.^62^ propose that a critical diameter exists at which solid particles liquefy and that the inverse of the critical diameter linearly increases with the temperature of the bulk phase transition.

The above body of literature does not include a clear methodology to predict the influence of particle size on the melting point, the glass transition temperature, and the temperature and humidity dependence of particle viscosity. Such predictions are needed to model the phase state of atmospheric particulate matter. This work presents a computationally inexpensive framework that is suitable for treating the size dependence of particle phase state. Available literature data are reviewed to constrain the model. Predictions are made for SOA generated from $\alpha$-pinene. Parameters that are important to the prediction of size dependent phase state are identified. Critical needs for future measurements characterizing the size dependence of phase transitions are described.

## Model

### Melting-point depression

The melting point depression for a spherical particle is computed using the Gibbs–Thomposon relationship^63^

1 $$\begin{aligned} \Delta T=\frac{6\sigma _{l/s}v_{s,m}}{D\Delta _{m}S} \end{aligned}$$

where *T* is the temperature, $\Delta T=(T_{bulk}-T)$ is the melting point depression defined as the difference between the melting point temperature of the bulk material and actual melting temperature, *D* is the particle diameter, $\sigma _{l/s}$ is the liquid/solid interfacial tension, $v_{s,m}$ is the molar volume of solid, $\Delta _{m}S$ is the molar entropy of the phase change at the melting temperature. The scaling of the influence of curvature, given by the prefactor 6 in Eq. 1, differs between different authors^63^. Equation 1 can be parameterized as

2 $$\begin{aligned} \Delta T=\xi D^{-1} \end{aligned}$$

where $\xi =6\sigma _{s/l}v_{s,m}/\Delta _{m}S$ is a parameter that subsumes the prefactor, the unknown physicochemical properties and depends on the particle composition.

**Figure 1:**
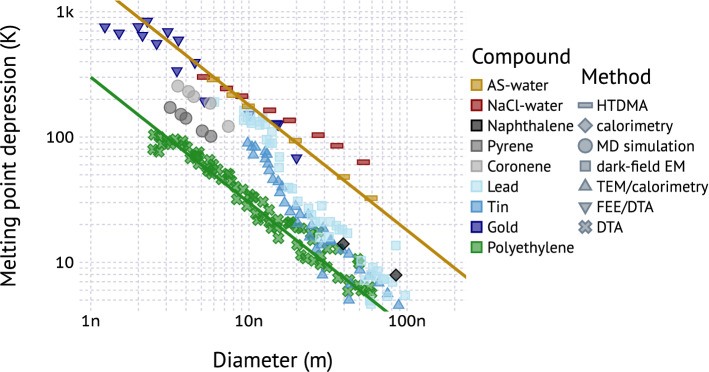
Summary of literature data for the size melting point depression versus size. Colors indicate the chemical composition groups: aqueous salt solutions (red/gold), polycyclic aromatic hydrocarbons (black/gray), metal particles (blue), polyethlene (green). Shapes denote the method used to generate the data. *HTDMA* humidified tandem differential mobility analyzer^52,55^, *MD* molecular dynamics simulation^66^, *(T)EM* (transmission) electron microscopy^67,68^, *FEE* field-electron emission^69^, *DTA* differential thermal analysis^70^. Solid lines correspond to Eq. 2 with $\xi =1800\,\hbox {K}\,\hbox {nm}$ (salmon) and $\xi =300\,\hbox {K}\,\hbox {nm}$ (green). Data sources are listed in the methods section.

Figure 1 summarizes available literature data of melting point depression for various composition categories. The data are obtained from a range of experimental techniques and include some molecular dynamic simulations. The data are approximately consistent with the Gibbs–Thompson slope of $-\,1$ in log–log space for the majority of the data, with obvious deviations for lead, tin, and aqueous NaCl. The deviations from Gibbs–Thompson for led and tin below 10 nm have been noted before^64,65^. Reasons for the deviation from Gibbs–Thompson include variation of surface tension and entropy of melting with size, as well as particle shape effects^65^. There is significant variability between different compounds. For example, a melting point depression of 30 K is required to liquify 10 nm polyethylene while 200 K is required for aqueous ammonium sulfate, lead or gold particles. There is no obvious clustering of broad compound classes, i.e. metals, inorganic salts, and organics. Nonetheless, the Gibbs–Thompson slope of $-\,1$ in log–log space is a reasonable approximation and there appears to be a limited range of $\xi$ values, despite a large variability of bulk melting point temperature (414–1337 K) for the graphed compounds. For the data shown, $\xi$ varies between 300 and 1800 K nm.

### Glass-transition and temperature-dependence of viscosity

The glass transition temperature is related to the bulk melting temperature via $T_{g}=gT_{m}$, which is known as the Boyer–Kauzmann rule. The value of *g* is $0.7\pm 0.2$ (supplemental information). It follows that the size dependence of the glass transition temperature is

3 $$\begin{aligned} T_{g}=T_{g}^{bulk}-g\xi D^{-1} \end{aligned}$$

where $T_{g}^{bulk}$ is the glass transition temperature for the bulk material. To the authors’ knowledge, no data on the melting point depression of amorphous glassy material is available. Equation 3 is therefore an untested hypothesis that will need to be validated against experimental data in future studies.

**Figure 2:**
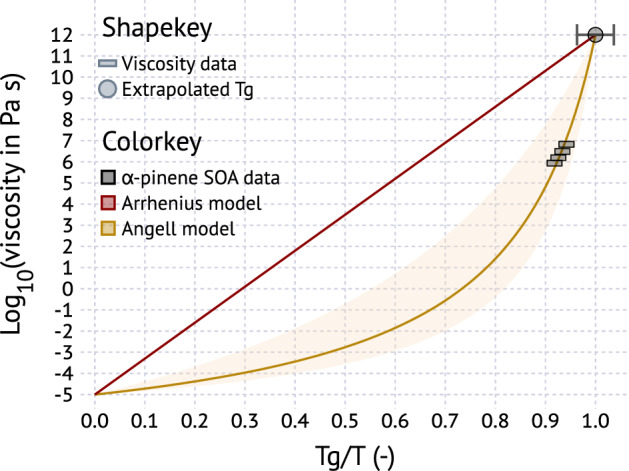
Angell^71^ representation of viscosity scaled by $T_{g}$. Viscosity and $T_{g}$ data are from Petters et al.^40^. The error bar on $T_{g}$ correspond to $\pm 10\,\hbox {K}$ uncertainty. The solid golden line and shaded area correspond to $D_{A}=7$ and $5<D_{A}<20$, respectively.

At temperatures warmer than $T_{g}$, but colder than $T_{m}$, viscosity decreases over many orders of magnitude. Angell^71,72^ showed that viscosity scaled by $T_{g}$ can be modeled using a simple parameterization

4 $$\begin{aligned} \log _{10}\eta =-5+0.434\left( \frac{39.17D_{A}}{D_{A}T/T_{g}+39.17T/T_{g}-39.17}\right) \end{aligned}$$

where $\eta$ is the viscosity and $D_{A}$ is the fragility parameter. Figure 2 illustrates the Angell representation of viscosity. At $T_{g}/T=1$ the particle is a glass and viscosity is taken to be $10^{12}\,\hbox {Pa}\,\hbox {s}$. At $T>T_{g}$, viscosity decreases. At very high temperature the viscosity approaches that of a gas, $\sim 10^{-5}\,\hbox {Pa}\,\hbox {s}$. The Arrhenius representation of the temperature dependence of viscosity corresponds to linear relationship in log-linear coordinates. Compounds following the Arrhenius law are dubbed “strong” glasses^71^. Fragile glasses deviate from the Arrhenius law and are described by Eq. 4. Shiraiwa et al.^32^ estimate $5<D_{A}<20$ for typical compounds found in SOA. Only very limited data on the temperature dependence of viscosity for SOA are available^40^. The glass-transition temperature in that work is from an extrapolation of temperature-dependent viscosity data. Extrapolated $T_{g}$ from that method agrees within $\sim \pm 10\,\hbox {K}$ of $T_{g}$ obtained from scanning calorimetry for citric acid and sucrose^44,45^. The fragility parameter for $\alpha$-pinene SOA is consistent with $D_{A}=7$. Importantly, aqueous solutions of sucrose may be described using a single fragility parameter^73^. This provides some justification to apply $D_{A}=7$ to dry SOA and aqueous solutions of SOA that form due to hygroscopic growth at elevated relative humidity.

### Humidity-dependence

Humidity dependence is modeled as described in Rothfuss and Petters^44^. $T_{g}$ of the binary solute and water system is generally is estimated using the Gordon–Taylor^74^ mixing rule^16,22,31,32,44,45,75–77^.

5 $$\begin{aligned} T_{g} =\frac{[1-w_{s}(RH)]T_{g,w}+\frac{1}{k_{GT}}w_{s}(RH)T_{g,s}}{1-w_{s}(RH)+\frac{1}{k_{GT}}w_{s}(RH)} \end{aligned}$$

6 $$\begin{aligned} w_{s}=\left( 1.0+\kappa _{m}\frac{a_{w}}{1-a_{w}}\right) ^{-1} \end{aligned}$$

where $w_{s}$ is the weight fraction of solute, $a_{w}$ is the water activity ($a_{w}=0.01RH\exp (A/D)^{-1}$, $A=8.69251 \times 10^{-6}\sigma _{s/a}/T$^78^, $\sigma _{s/a}$ is the surface tension, RH is the relative humidity in %), $\kappa _{m}$ is the mass based hygroscopity parameter^79^, $T_{g}$ is the glass transition temperature of the mixture, $T_{g,w}$ the glass transition temperature of water, $T_{g,s}$ is the glass transition temperature of the solute and $k_{GT}$ is the Gordon–Taylor constant. In contrast to the formulation described in Rothfuss and Petters^44^, Eq. 6 accounts for the effect of curvature on water content.

The Gordon–Taylor mixing rule has its origin in polymer science and for some polymer systems the Gordon–Taylor constant can obtained from the density and expansion coefficient. This however fails when applying the rule to non-polymers and systems containing water. In such systems $k_{GT}$ becomes an empirical coefficient associated with the compound. A number of expressions having similar mathematical form have been proposed^80^. Variations of the mixing rule include the Fox^81^ equation, the Couchman and Karasz^82^ equation, which is as the Gordon–Taylor rule but using mole fraction as composition variable and heat capacity ratios to express *k*, and the Kwei^83^ equation, which is as the Gordon–Taylor rule but also includes higher order terms. Secondary organic aerosol is a mixture comprising 100 s of components^84,85^. One approach is to group the organic compounds together and then use a binary Gordon–Taylor mixing rule to treat mixtures of water and the lumped organic fraction^86^, which is denoted as the quasi-binary assumption.

Here the Gordon–Taylor mixing rule is applied to SOA using the quasi-binary assumption with the following inputs. The glass transition temperature for $\alpha$-pinene-derived SOA is $T_{g,s}^{bulk}=271.7\pm 10\, \hbox {K}$^40^. The $\pm 10\, \hbox {K}$ estimate denotes the accuracy of a single measurement and does not account for composition differences that arise from differences in SOA generation method between studies. Furthermore, $T_{g,w}=136 \hbox {K}$^31^, $k_{GT}=2.5\pm 1.5$^22,31,87^ and $\kappa _{m}=0.04\pm 0.035$. The range $\kappa _{m}=0.04\pm 0.035$ neglects the potential systematic variation of $\kappa _{m}$ with relative humidity. Further details are provided in the methods and supplemental information.

### Viscosity model

Let the generic function definitions $T_{g}^{size}(T_{g}^{bulk},D)$ denote Eq. 3, $T_{g}^{bulk}(w_{s}(RH),T_{g,s}^{bulk})$ denote Eq. 5, $w_{s}(RH$) denote Eq. 6, and $\eta _{A}(T,T_{g})$ denote Eq. 4. Viscosity as a function of *T*, RH, and *D* is computed via function composition.

7 $$\begin{aligned} \eta (T,RH,D)=\eta _{A}\left( T,T_{g}^{size}(T_{g}^{bulk}(w_{s}(RH),T_{g,s}^{bulk}),D)\right) \end{aligned}$$

where the composite function $\eta (T,RH,D)$ also depends on the solute parameters $T_{g,s}^{bulk}=271.7\pm 10K$, $\kappa _{m}=0.04\pm 0.035$, $k_{GT}=2.5\pm 1.5$, $D_{A}=7$, $g=0.7$ and $\xi =1050\pm 750\, \hbox {K nm}$; the values are estimates for $\alpha$-pinene SOA, except for the range in $\xi$, which is based on the data in Fig. 1. In the limit of infinitely large particles, this model is identical to the model of DeRieux et al.^87^. However, the solute parameters differ from DeRieux et al.^87^ due to newly available experimental constraints on fragility and different assumptions made about hygroscopicity. A detailed representation of Eq. 7 is given in the supplemental information.

## Results and discussion

**Figure 3:**
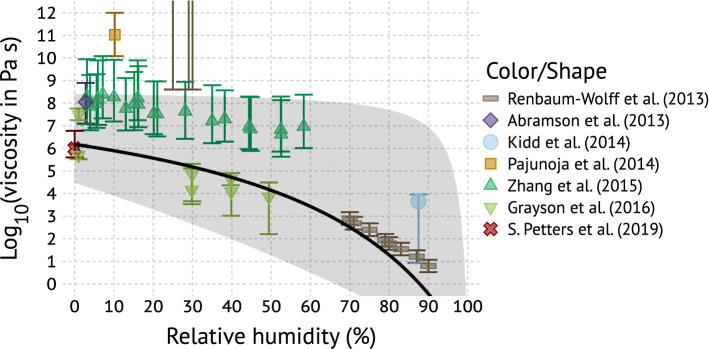
Measured^27,30,40,88–91^ and predicted viscosity of $\alpha$-pinene SOA at room temperature as a function of relative humidity. The measurements by Renbaum-Wolff et al.^30^ at RH 20-30% correspond to a lower limit estimate of their poke-flow measurements (horizontal bar) with possible values being up to, or exceeding $10^{12}\,\hbox {Pa}\,\hbox {s}$. The solid line and gray shaded area corresponds to $T_{g,s}^{bulk}=271.7\pm 10\,\mathrm{K}$, $\kappa _{m}=0.04\pm 0.035$, $k_{GT}=2.5\pm 1.5$, $g=0.7$, and $D_{A}=7$.

Figure 3 shows measured and predicted viscosity of $\alpha$-pinene SOA at room temperature as a function of relative humidity without taking the potential size dependence of $T_{g}$ into account. The slope of the $\eta$-RH relationship is determined by $k_{GT}$ and $\kappa _{m}$. Data from Pajunaja et al.^90^ and Renbaum-Wolff et al.^30^ show viscosity $>10^{9}\,\hbox {Pa}\,\hbox {s}$ at $\hbox {RH} < 30\%$ and fall outside the shaded area. Increasing the range in $T_{g,s}^{bulk}$ to $\pm 25\,\hbox {K}$, as was assumed in DeRieux et al.^87^, would include those points inside the shaded area. $T_{g,s}$ depends on molecular weight and functional group composition and it small differences in these parameters have a large effect on $T_{g,s}$. Thus composition differences between the studies likely explain the higher viscosity in these experiments^40^. Overall the model and parameter ranges are in reasonable agreement with most of the published data. The remainder of this work will use the parameter ranges $T_{g,s}^{bulk}=271.7\pm 10\,\mathrm{K}$, $\kappa _{m}=0.04\pm 0.035$, $k_{GT}=2.5\pm 1.5$, $D_{A}=7$, $g=0.7$ and $\xi =1050\pm 750\,\hbox {K}\,\hbox {nm}$ to explore the dependence of viscosity on particle size.

**Figure 4:**
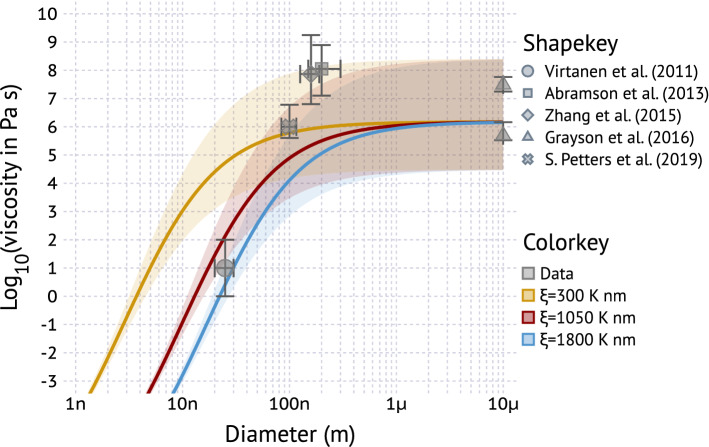
Measured and predicted viscosity of $\alpha$-pinene SOA at room temperature as a function of particle diameter. The solid lines and shaded area corresponds to predicted viscosity assuming $T_{g,s}^{bulk}=271.7\pm 10\,{{\rm K}}$, $\kappa _{m}=0.04\pm 0.035$, $k_{GT}=2.5\pm 1.5$, $D_{A}=7$, $g=0.7$, and RH = 0%. Colors delineate different values of $\xi$ spanning the range of values shown in Fig. 1.

Figure 4 shows the measured and predicted viscosity of $\alpha$-pinene SOA at room temperature as a function of particle diameter. Data are as the same as in Fig. 3 for the lowest RH reported but plotted at the measurement diameter. The Grayson et al.^91^ are obtained using a “bulk” poke-flow method and are plotted at $10 \, \upmu \hbox {m}$ diameter for reference. Note that the Virtanen et al.^61^ data differ in several respects. The data are taken at RH $=$ 31%, significantly higher than the other values (0–2%). The SOA is from oxidation of pine emitted VOCs and not pure $\alpha$-pinene and the data are not direct measurements of viscosity. Virtanen et al. observed a decrease in rebound fraction in an impactor for $D<30\,\hbox {nm}$. Upon impaction, a fraction of the energy is dissipated due to particle deformation (i.e. viscosity) while the remainder is available for rebound. Rebound occurs when the kinetic energy exceeds the energy adhesion^61^. Virtanen et al. show that the predominant impaction energy of the smaller particles is larger than the impaction energy of the larger particles, and thus that the decreasing rebound fraction indicates a decrease in viscosity^36,92^. Assigning a viscosity to the Virtanen data in retrospect is difficult. Bateman et al.^36^ calibrated a different impactor at a different size with sucrose and report that the viscosity range for that transition is 1–100 Pa s. That value range is expected to depend on the impactor surface properties and flow dynamics. Slade et al.^41^ used a low pressure impactor similar in design to that in Virtanen et al. and classify particles with a rebound fraction $< 0.2$ as “liquid” while assigning “liquid” a viscosity $< 100\,\hbox {Pa}\,\hbox {s}$. Therefore, the Virtanen et al.^61^ observations, which show bounce fraction $< 0.2$ at $\sim 25 \,\hbox {nm}$, are graphed in Fig. 4 having viscosity 1–100 Pa s. Future measurements are needed to confirm that value.

As expected, the predicted dependence of viscosity on particle size depends on $\xi$, with larger values of $\xi$ corresponding to a larger decrease in viscosity with decreasing size. The data are broadly consistent with $1050<\xi <1800\,\hbox {K}\,\hbox {nm}$. The exact value remains unclear due to the need to juxtapose data from different sources. This results in an the imperfect match of relative humidity. There are also differences in chemical composition due to differences in mass loading, oxidant exposure, size-dependence of composition, aerosol preparation method and aerosol age, all of which may affect the glass transition temperature and viscosity. Finally, only a single data point at $D<100\,\hbox {nm}$ is available. This point is also taken at elevated relative humidity. If humidity influences the observed viscosity, the viscosity under dry conditions would have been higher, which in turn would alias as $\xi <1050$. Due to the very limited available aerosol viscosity data a preliminary range $300<\xi <1800\,\hbox {K}\,\hbox {nm}$ for organic aerosol is proposed, which is based on the survey presented in Fig. 1. Systematic studies of the effect of particle size on viscosity are needed, including the study of pure compounds and of complex mixtures such as SOA.

The model predictions show a decrease in viscosity to that of a thin liquid, defined as $<10^{-3}\,\hbox {Pa}\,\hbox {s}$ at 1.6, 5.5, and 9.3 nm for $\xi =300$, 1050, and 1800 K nm, respectively. Changing relative humidity or temperature in the model changes the viscosity at large size, but it has a negligible effect on the slope of viscosity vs. size at small diameter and the size of the intersection with $10^{-3}\,\hbox {Pa}\,\hbox {s}$. The uncertainty in the *g* value from application of the Boyer–Kauzmann rule ($g=0.7\pm 0.2$) slightly alters the intersection with $10^{-3}\,\hbox {Pa}\,\hbox {s}$. Sensitivity analysis to these parameters is provided in the supplemental information. The predicted decreased below $10^{-3}\,\hbox {Pa}\,\hbox {s}$ implies that the mixture is behaving more like a gas and is equivalent to volatilization due to decreasing particle curvature and increasing Kelvin effect.

The model formulated in Eq. 7 only relies on simple algebraic functions. It is computationally efficient and thus suitable for inclusion in atmospheric models. Input parameters such as $T_{g/s}$ and $\kappa _{m}$ can be predicted from various proxies for organic aerosol composition^32,87,93–95^. However, prediction of the fragility parameter ($D_{A}$), Gordon–Taylor constant ($k_{GT}$) and $\xi$ from composition data are not yet available. Nevertheless, reasonably constrained ranges for these parameters exist and have been identified here or in previous studies. The parameter $\xi =6\sigma _{s/l}v_{s,m}/\Delta _{m}S$ can be predicted from first principles provided $\sigma _{s/l}$, $v_{s,m}$, and $\Delta _{m}S$ data are available. For example, crystalline pyrene has $\sigma _{s/l}=22.8\times 10^{-3}\,{\hbox {J}\,\hbox {m}}^{-2}$ (ref.^96^), $v_{s,m}=1.59\times 10^{-4} \, {\hbox {m}}^{3}\,{\hbox {mol}}^{-1}$ (based on molecular weight and density), and $\Delta _{m}S=40.97 \, {\hbox {J mol}}^{-1} \, {\hbox {K}}^{-1}$ (ref.^97^). Therefore $\xi =6\sigma _{s/l}v_{s,m}/\Delta _{m}S=531\, \hbox {K nm}$ is predicted. A fit to the pyrene data from MD simulations in Fig. 1 yields 560 K nm. Systematic molecular dynamics simulations^66,98^ systematic evaluation of available surface tension and entropy of melting data may provide additional constraints on $\xi$ and its dependence on composition.

## Conclusions

Data of the melting point depression as a function of particle size have been reviewed. A parameter $\xi$ is abstracted from the Gibbs–Thompson relationship, where $\xi$ characterizes the size dependence of the melting point for a fixed particle composition. The parameterization is applied to predict changes in glass transition temperature and viscosity as a function of size. Only limited aerosol data are available to validate the model. From these data a preliminary range $300<\xi <1800\,\hbox {K}\,\hbox {nm}$ for organic aerosol is proposed. New experimental data of the size-dependence of particle viscosity for atmospheric aerosol mimics are needed to thoroughly validate the predictions made herein. The model framework, together with the identified parameter ranges, can be used to evaluate the potential influence of melting point depression on atmospheric processes.

## Methods

### Melting-point depression

Melting point depression data collated in Fig. 1 were digitized from data displayed in figures in the literature. Details about the digitization method are given in the supplemental information. The digitized data plotted are summarized in a comma delineated text file that contains the melting point depression ($\Delta T$), particle diameter (*D*), the source citation, the source figure that was digitized, and the method that was used to generate the data. Tin data are taken from Fig. 2 in Koppes et al.^99^ including data from Wronski^68^ and Lai et al.^100^. Gold data correspond to spherical gold particles summarized in Fig. 2 by Lu et al.^101^ with data from Castro et al.^69^ and Dick et al.^102^. Lead data are taken from Fig. 2 in David et al.^67^. Pyrene and coronene data are taken from Fig. 5 in Chen et al.^98^; naphthalene data are taken from Fig. 4 in Jackson and McKenna^103^. Polyethylene data is from Fig. 1 in Jiang et al.^66^ including data for linear alkanes^104^, cyclic alkanes^105^, and chain-extended polyethylene^70^. Data for aqueous salt solutions is taken from Fig. 5 in Cheng et al.^62^ with data derived from Biskos et al.^52,55^.

### Hygroscopicity data

Volume-based hygroscopicity parameter^106^ data for $\alpha$-pinene SOA were digitized from data displayed in figures and tables in the literature^38,107,108^. The digitized data plotted are summarized in a comma delineated text file that contains relative humidity, $\kappa _{v}$ value, and the source citation. Volume-based hygroscopicity parameter^106^ data are converted to mass-based hygroscopicity^79^ using

8 $$\begin{aligned} \kappa _{m}=\kappa _{v}\frac{\rho _{w}}{\rho _{s}} \end{aligned}$$

where $\kappa _{m}$ is the mass-based hygroscopicity parameter^79^, $\rho _{w}$ and $\rho _{s}$ are the density of water and solute, respectively. The density of $\alpha$-pinene SOA in these studies is unknown. Kuwata et al.^109^ report $1.23<\rho _{s}<1.46\,{\hbox {g}\,\hbox {cm}}^{-3}$ for $\alpha$-pinene SOA. The uncertainty due to unknown density in the conversion from $\kappa _{v}$ to $\kappa _{m}$ is unimportant relative to the reported range in values. The data are graphed in the supplemental information.

### Viscosity data

Viscosity data collated in Figs. 2, 3 and 4 were digitized from data displayed in figures and tables in the literature^27,30,40,88–91^. The digitized data plotted are summarized in comma delineated text files. Viscosity data points in Fig. 2 were generated as follows. The logistic fit to the particle shape parameter measurement for $\alpha$-pinene (supplemental information ref.^40^) was solved for the interval $[x_{0}-\sigma ,x_{0}+\sigma ]$, where $x_{0}$ corresponds to the mean and $\sigma$ to the slope of the transition, and $x_{0}=20.2\,^{\circ }\mathrm{C}$ and $\sigma =4.93$ for $\alpha$-pinene. The interval was discretized into $2.5\,^{\circ }\mathrm{C}$ steps. The interval only determines the density of points plotted in Fig. 2. The shape parameter was converted to viscosity using sintering theory^25,110^ and the temperature was normalized by the $T_{g}$ extrapolated from the same measurements.

## Supplementary information

Supplementary Information.

## Data Availability

All data and scripts used to create the figures in this manuscript are freely available through GitHub and DockerHub. Data and scriptsare archived in an online data repository at 10.5281/zenodo.3824214.). Further details are in the supplemental information.
