# Supplementary material for: Predicting the influence of particle size on the glass transition temperature and viscosity of secondary organic material
Source: Sci Rep. 2020 Sep 16;10:15170. doi: 10.1038/s41598-020-71490-0 (PMC7495436; doi:10.1038/s41598-020-71490-0)
Supplement: Supplementary file 1 — Supplementary Information. [file 41598_2020_71490_MOESM1_ESM.pdf]

Supplement of
Predicting the influence of particle size on the glass transition temperature and viscosity of secondary organic material

Markus Petters[1,*] **and Sabin Kasparoglu**[1]

[1]NC State University, Department of Marine, Earth, and Atmospheric Sciences, Raleigh, 27695-8208, USA
[*]mdpetter@ncsu.edu

Mass-based hygroscopicity parameter

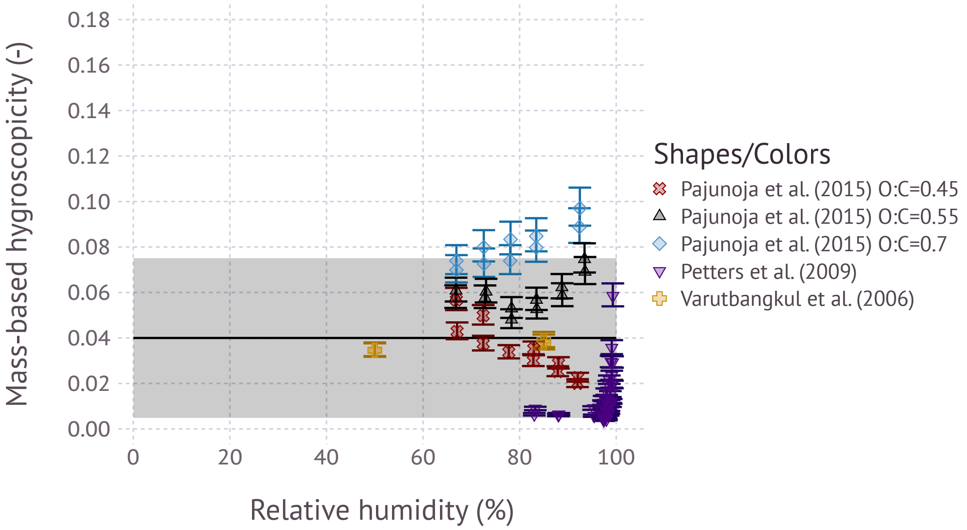

Figure S1. Summary of literature data for mass-based hygroscopicity parameter. Shapes/Colors indicate different sources: *Pajunoja et al.*[1], *Petters et al.*[2], and *Varutbangkul et al.*[3]. The data from *Petters et al.* include their Type I, II, and III data, which have slightly different oxidation state. The solid black line and gray shaded area correspond to $\kappa_m = 0.04 \pm 0.035$. Error bars denote the uncertainty due to unknown density in the κ_v-to-κ_m conversion and represent the range $1.23 < \rho_s < 1.46$[4].

Figure S1 summarizes the estimated κ_m from literature data. In general, κ_m depends on the atomic O:C ratio (or the degree of oxidation), the molecular weight, and may vary systematically with relative humidity. Both increasing and decreasing κ with decreasing RH are observed. There is a wide range of observed values. The uncertainty due to unknown density in the κ_v-to-κ_m is much smaller than the spread in the data, and the trends within each data set. No data below 50% RH are available, which is in part due to the small water content present in the highly viscous states. The data can be broadly summarized as $\kappa_m = 0.04 \pm 0.035$.

Boyer–Kauzmann Rule

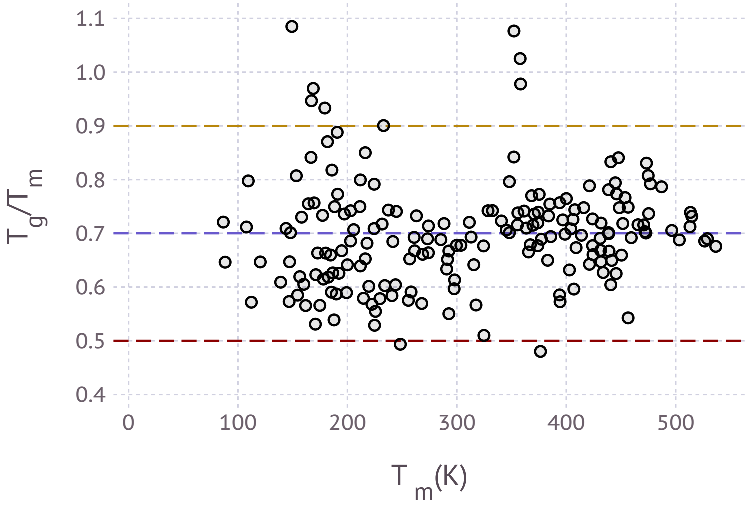

Figure S2. Summary of literature data for the ratio of T_g/T_m for single compounds, stratified by temperature.

The Boyer–Kauzmann rule states that $T_g = gT_m$. Figure S2 summarizes T_g and T_m data taken from Figure A2 in DeRieux et al.[5]. The data show that the majority of compounds can be described using $g = 0.7 \pm 0.2$.

Model Sensitivity

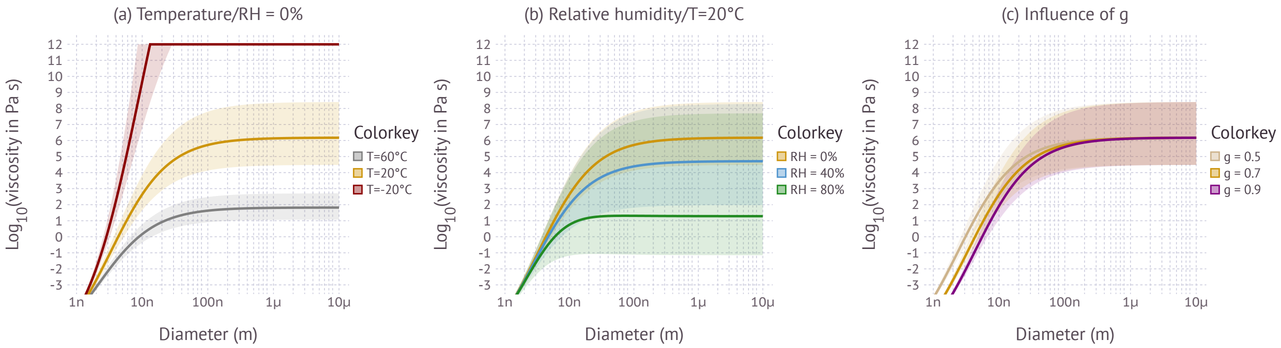

Figure S3. Predicted viscosity of α-pinene SOA as a function of particle diameter. The solid lines and shaded area corresponds to predicted viscosity assuming $T_{g,s}^{bulk} = 271.7 \pm 10K$, $\kappa_m = 0.04 \pm 0.035$, $k_{GT} = 2.5 \pm 1.5$, $D_A = 7$, $g = 0.7$ and $\xi = 300$ K nm. (a) Colors delineate different values of T at RH = 0%. (b) Colors indicate different values of RH at T = 20°C, (c) colors indicate different values of g at RH=0% at $T = 20°C$.

Figure S3 summarizes the model sensitivity to temperature, relative humidity, and the g value.. The $T = 20°C$/RH = 0% prediction is identical to Figure 2 in the main manuscript. At large size, decreasing the temperature by 40°C results in glassy conditions. Conversely, increasing the temperature decreases the viscosity. These are in accordance with the fragility parameterization. Increasing relative humidity decreases the viscosity at large size, in accordance with humidity dependence shown in Figure 3 in the main manuscript. The intersection point with 10^{-3} Pa s is hardly affected by the assumed T/RH and weakly affected by the assumed g.

Data Digitization

All data were digitized using the “DataThief III” software. The software allows the user to place axis markers and select the axis scaling. The user then places a marker on top of the original marker. Errors in the digitization process include (1) placement

of the axis markers, (2) placement of the marker on the marker symbol, and (3) potential errors associated with the plotting method/plotting software used by the authors. Assuming that the original graph is a faithful representation of the data, and using readouts to test accuracy along a single axis, we estimate that the digitization error is much smaller than the uncertainty in the individual measurements.

Summary of Model Equations

Below is a linear description of the size-dependent model as given in the manuscript.

$$w_s(RH) = \left(1.0 + \kappa_m \frac{0.01RH\exp(A/D)^{-1}}{1-0.01RH\exp(A/D)^{-1}}\right)^{-1} \tag{1}$$

$$T_g^{bulk}(w_s(RH), T_{g,s}^{bulk}) = \frac{[1-w_s(RH)]T_{g,w} + \frac{1}{k_{GT}} w_s(RH)T_{g,s}}{1-w_s(RH) + \frac{1}{k_{GT}} w_s(RH)} \tag{2}$$

$$T_g^{size}(T_g^{bulk}, D) = T_g^{bulk} - g\xi D^{-1} \tag{3}$$

$$\eta_A(T, T_g) = \exp_{10}\left(-5 + 0.434\left[\frac{39.17D_A}{D_A T/T_g + 39.17T/T_g - 39.17}\right]\right) \tag{4}$$

$$\eta(T, RH, D) = \eta_A(T, T_g^{size}(T_g^{bulk}(w_s(RH), T_{g,s}^{bulk}), D)) \tag{5}$$

where, η is the viscosity, T is temperature, RH is the relative humidity [%], D is the particle diameter, g is the ratio from the Boyer-Kauzmann rule, ξ is a parameter that describes the sensitivity to particle size, $T_{g,s}^{bulk}$ is the bulk glass transition temperature of the solid, $T_{g,w}$ is the glass transition temperature of water, $T_{g,s}^{size}$ is the glass transition temperature of particle with diameter D, w_s is the weight fraction of the solute, κ_m is the mass-based hygroscopicity parameter, $A = 8.69251\times10^{-6}\sigma_{s/a}/T$, $\sigma_{s/a}$ is the surface tension, and D_A is the fragility parameter.

Data Availability

GitHub

The data and software scripts are currently available in a publicly accessible GitHub repository: https://github.com/mdpetters/predictingInfluenceParticleSize2020. Data are stored in comma delineated ASCII files. Scripts generating the figures in the manuscript are written in Julia version 1.4.0. The computing environment is specified in the Dockerfile, Project.toml, and Manifest.toml. An easy means to run and visualize the scripts is through the Jupyter notebook "Supplement.ipynb".

Docker Container

A Docker container is available through DockerHub. The docker container is a virtual machine that contains all software and dependencies needed to execute the code. It can be loaded through the command

```
docker run -it -p 8888:8888 mdpetters/predicting_influence_particle2020:final
```

which will download the image from the repository and execute it. The freely available Docker engine must be installed on the local computer (https://docs.docker.com/install/). The command produces the output shown in Figure S4. Copying the displayed web address from the loaded session

```
http://127.0.0.1:8888/?token=0ac7fee4e87fabf7fbb0577826712541d5cb85cb82621beb
```

will start the Jupyter notebook server in the local browser. From there the Jupyter notebook Supplement.ipynb can be loaded and executed. The code in the individual scripts can be altered during the session.

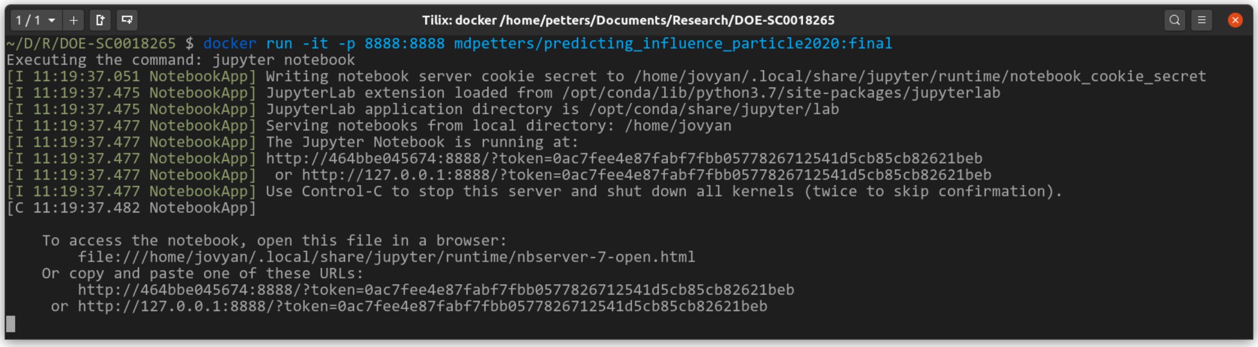

Figure S4. Terminal output of the docker command

Archive

The GitHub files and Docker container are archived in an online data repository at https://doi.org/10.5281/zenodo.3824214.

References

1. Pajunoja, A. *et al.* Adsorptive uptake of water by semisolid secondary organic aerosols. *Geophysical Research Letters* **42**, 3063–3068 (2015).
2. Petters, M. D. *et al.* Towards closing the gap between hygroscopic growth and activation for secondary organic aerosol - Part 2: Theoretical approaches. *Atmospheric Chemistry and Physics* **9**, 3999–4009 (2009).
3. Varutbangkul, V. *et al.* Hygroscopicity of secondary organic aerosols formed by oxidation of cycloalkenes, monoterpenes, sesquiterpenes, and related compounds. *Atmos. Chem. Phys.* **6**, 2367–2388 (2006).
4. Kuwata, M., Zorn, S. R. & Martin, S. T. Using Elemental Ratios to Predict the Density of Organic Material Composed of Carbon, Hydrogen, and Oxygen. *Environmental Science & Technology* **46**, 787–794 (2012).
5. DeRieux, W.-S. W. *et al.* Predicting the glass transition temperature and viscosity of secondary organic material using molecular composition. *Atmos. Chem. Phys.* **18**, 6331–6351 (2018).
